# Relation of Sleep Quality to a Panel of Plasma Cardiometabolic Markers in Airline Pilots: A Cross-Sectional Study

**DOI:** 10.7759/cureus.51650

**Published:** 2024-01-04

**Authors:** Miryam Liaño Riera, Andrés Santiago Sáez, Ángel García Martín, Manuel Gómez Serrano, Piercarlo Minoretti

**Affiliations:** 1 Legal Medicine, Psychiatry, and Pathology, Complutense University of Madrid, Madrid, ESP; 2 Legal Medicine, Hospital Clinico San Carlos, Madrid, ESP; 3 General Direction, Studio Minoretti, Oggiono, ITA

**Keywords:** growth differentiation factor-15, fibroblast growth factor-21, adiponectin, biomarkers, pittsburgh sleep quality index, sleep quality, airline pilots

## Abstract

Background

Insomnia and poor sleep are leading modifiable risk factors for cardiovascular disease. Given the high susceptibility of airline pilots (APs) to sleep disturbances, we sought to investigate the hypothesis that poor sleep in this professional group correlates with alterations in plasma biochemical markers that would reflect critical aspects in the pathophysiology of cardiometabolic disorders.

Methods

In this preliminary cross-sectional study, we examined the relation of poor sleep to fourteen plasma biomarkers reflecting multiple cardiometabolic pathways in a convenience sample of 117 male APs. The Pittsburgh Sleep Quality Index (PSQI) was used to categorize the participants into good sleepers (n = 70, 59.8%; PSQI scores from 0 to 4) and poor sleepers (n = 47, 40.2%; PSQI scores of 5 or higher). The concentrations of biomarkers were compared between the two groups using both univariable and multivariable analyses.

Results

Compared to good sleepers, APs identified as poor sleepers exhibited significantly different levels of four plasma cardiometabolic biochemical markers in univariable analysis. However, in multivariable-adjusted analysis, only three biomarkers, adiponectin, fibroblast growth factor (FGF)-21, and growth differentiation factor (GDF)-15, remained independently associated with poor sleep.

Conclusion

Poor sleep quality in APs correlates with lower plasma concentrations of adiponectin and elevated levels of FGF-21 and GDF-15. Further longitudinal studies are required to elucidate the role of these biomarkers in the link between sleep disturbances and cardiometabolic risk in this professional group.

## Introduction

The prevalence of sleep disturbances among airline pilots (APs) is notably higher than in the general population, with research indicating that between 34.9% and 59.3% of pilots experience disrupted sleep and daytime sleepiness [1]. Despite efforts to enhance sleep health within this profession, these issues persist [2]. Pilots are also more susceptible to developing obstructive sleep apnea (OSA), with reported prevalence rates ranging from 17.2% [3] to a staggering 70.9% [4]. Furthermore, unintentional sleep during flight, a situation that could potentially jeopardize passenger safety, has been reported in up to 57.8% of pilots [5]. These figures underscore the urgent need for effective strategies to address sleep health in the aviation industry.

Inadequate sleep in the general population confers a higher risk of adverse cardiovascular outcomes, irrespective of traditional risk factors [6, 7]. Given the high susceptibility of APs to sleep disturbances, it is unsurprising that this professional group has a reported increased risk of cardiometabolic diseases [8]. However, the exact pathophysiological mechanisms linking disrupted sleep and poor cardiovascular health in APs remain unclear. Numerous circulating biomarkers have been associated with cardiometabolic diseases in the general population [9]. However, their study in pilots is limited and, to our knowledge, has not been previously examined in relation to disrupted sleep in this professional category. Identifying the associations between plasma biochemical markers, which could reflect critical aspects of cardiometabolic disorders' pathophysiology [10], and poor sleep quality in APs may provide valuable insights into the pathogenesis of their increased cardiometabolic risk. This knowledge could also serve as a foundation for identifying APs who are particularly susceptible to developing sleep disturbances, thereby enabling the early implementation of preventive or corrective measures.

Building on these premises, we carried out a preliminary cross-sectional study to measure plasma levels of 14 distinct cardiometabolic biomarkers, representative of various pathophysiological pathways, including adipokines, markers of inflammation, endothelial function, and cardiomyocyte injury, within a cohort of APs. We categorized the study participants based on the presence or absence of poor sleep quality to investigate the differences in plasma levels of cardiometabolic biomarkers. Our findings collectively have the potential to reveal suggestive pathophysiological links between sleep disruption and cardiovascular consequences in this professional group.

## Materials and methods

Participants

This study is part of an ongoing project aimed at identifying potential biomarkers associated with poor sleep in APs [11]. We analyzed a convenience sample of 117 male APs of Caucasian descent who voluntarily participated in the study. All pilots were recruited during their regular occupational health visits at outpatient clinics (Studio Minoretti SRL, Oggiono, Italy), where they were invited to participate by an experienced occupational health physician. The study was limited to male subjects due to the low number of female pilots. We excluded individuals with psychiatric, neurological, endocrine, infectious, autoimmune, or malignant diseases. APs with known OSA and those who had used any medication or dietary supplements within the previous three months were also deemed ineligible. All participating APs were found to be in satisfactory physical health. The study received approval from the local ethics committee (Studio Minoretti, reference number: 2021/04E), and all participants gave written informed consent.

Sleep quality

The Pittsburgh Sleep Quality Index (PSQI), a validated self-reported questionnaire, was employed to measure the sleep quality of airline pilots [12]. This instrument, comprising 19 items, is widely recognized in research for its robust psychometric properties. The questionnaire generates a global score, ranging from 0 to 21, where higher scores signify poorer sleep quality. A score above 5 typically indicates poor sleep quality [12]. The PSQI has shown a sensitivity of 89.6% and a specificity of 86.5% in detecting individuals with sleep disorders, using a standard cutoff score of 5 [13].

Laboratory methods

Venous blood samples were drawn while fasting, collected in ethylenediaminetetraacetic acid (EDTA)-containing tubes, and centrifuged at 1500 g for a duration of 15 minutes. The plasma obtained from this process was separated, divided into aliquots, and stored at a temperature of -80 °C until analysis. Laboratory personnel, who were not privy to any clinical data, conducted all immunoassays. The analysis encompassed fourteen circulating biomarkers, representative of various cardiometabolic pathways. These included adipokines, and markers of inflammation, endothelial function, and cardiomyocyte injury. The selected biomarkers were D-dimer, intercellular adhesion molecule-1 (ICAM-1), matrix metalloproteinase-9 (MMP-9), myeloperoxidase (MPO), N-terminal pro-brain natriuretic peptide (NT-proBNP), osteoprotegerin, osteopontin, soluble receptor for advanced glycation end-products (sRAGE), vascular cell adhesion molecule-1 (VCAM-1), fibroblast growth factor-21 (FGF-21), growth differentiation factor-15 (GDF-15), CD40 ligand (CD40L), monocyte chemoattractant protein-1 (MCP-1), and adiponectin. These molecules were chosen opportunistically based on their biological plausibility, clinical relevance, and the availability of commercially available enzyme-linked immunosorbent assays (ELISAs). All analytical procedures were performed in accordance with the guidelines provided by the respective kit manufacturers.

Data analysis

APs scoring between 0 and 4 on the PSQI were classified as good sleepers, whereas those scoring 5 or above were deemed poor sleepers [11]. The Kolmogorov-Smirnov test was utilized to evaluate the normality of continuous data. Variables with a normal distribution were represented as means ± standard deviations, while skewed variables were expressed as medians and interquartile ranges. For comparative purposes, the Student’s t-test was applied to normally distributed data, and the Mann-Whitney U test was used for skewed variables. Categorical variables were presented as counts and analyzed using the chi-square test. The Spearman’s correlation coefficient (rho) was employed to examine the univariate correlations between plasma markers and PSQI scores. The relationship between biomarkers (exposure) and the presence of poor sleep was initially investigated through univariable analysis. Cardiometabolic markers with a P-value <0.05 were further assessed using multivariable modeling, adjusted for potential confounders, to evaluate their independent association with poor sleep. Results were presented as odds ratios (ORs) with their corresponding 95% confidence intervals (CIs). Data analysis was performed using the IBM SPSS Statistics for Windows, Version 20, (Released 2011; IBM Corp., Armonk, New York, United States). A two-tailed P-value <0.05 was considered statistically significant. Due to the exploratory nature of the study, Bonferroni's correction was not applied.

## Results

Using the PSQI, APs (n = 117) were divided into good sleepers (n = 70, 59.8%; PSQI scores from 0 to 4) and poor sleepers (n = 47, 40.2%; PSQI scores of 5 or higher). Table 1 provides a detailed overview of the general characteristics of these two groups. Notably, there were no significant intergroup differences in terms of common cardiovascular risk factors.

**Table 1:** General characteristics of airline pilots categorized according to the quality of sleep Categorical data are expressed as counts and percentages, whereas continuous variables are presented as means ± standard deviations. ns, not significant

Characteristic	Good sleepers (n = 70)	Poor sleepers (n = 47)	P
Age, years	41 ± 5	40 ± 6	ns
Male sex, n (%)	70 (100)	47 (100)	ns
BMI, kg/m^2^	24 ± 4	23 ± 4	ns
Education, years	17 ± 4	17 ± 4	ns
Systolic blood pressure, mmHg	120 ± 20	121 ± 18	ns
Diastolic blood pressure, mmHg	74 ± 9	73 ± 10	ns
Diabetes mellitus, n (%)	0 (0)	0 (0)	ns
Current smoking, n (%)	8 (11.4%)	6 (12.8%)	ns

Among the fourteen candidate markers that were examined in plasma samples (Table 2), four, including adiponectin, sRAGE, FGF-21, and GDF-15, were found to differ significantly in the two groups in univariable analysis.

**Table 2:** Plasma cardiometabolic markers in airline pilots categorized according to the quality of sleep Normally distributed variables are presented as means ± standard deviations, whereas skewed variables were summarized by medians and interquartile ranges. ICAM-1, intercellular adhesion molecule-1; MMP-9, matrix metalloproteinase-9; MPO, myeloperoxidase; NT-proBNP, N-terminal pro-B-type natriuretic peptide; sRAGE, soluble receptor for advanced glycation endproducts; VCAM-1, vascular cellular adhesion molecule-1; FGF-21, fibroblast growth factor-21; GDF-15, growth differentiation factor-15; CD40L, CD40 ligand; MCP-1, monocyte chemoattractant protein-1; ns, not significant.

Biomarker	Good sleepers (n = 70)	Poor sleepers (n = 47)	P
D-dimer, µg/mL	0.2 (0.1–0.4)	0.2 (0.1–0.3)	ns
ICAM-1, ng/mL	499 (262–675)	502 (285–694)	ns
MMP-9, ng/mL	3.9 (1.9–5.3)	4.0 (1.7–5.5)	ns
MPO, ng/mL	11.2 (9.7–13.5)	11.8 (9.3–13.2)	ns
NT-proBNP, pg/mL	20.3 (8.8–28.7)	20.7 (8.6–27.6)	ns
Osteoprotegerin, pg/mL	851 (610–1186)	874 (677–1090)	ns
Osteopontin, ng/mL	29.2 (24.3–38.3)	29.7 (25.8–39.1)	ns
sRAGE, ng/mL	1.9 (0.9–3.4)	1.5 (0.6–2.9)	<0.05
VCAM-1, ng/mL	830 (625–1072)	837 (615–1083)	ns
FGF-21, ng/mL	3.5 (3.2–3.9)	4.3 (3.7–5.2)	<0.001
GDF-15, ng/mL	0.4 (0.2–0.6)	0.7 (0.4–0.9)	<0.001
CD40L, ng/mL	1.9 (1.4–2.2)	1.8 (1.5–2.1)	ns
MCP-1, pg/mL	288 (260–312)	295 (271–320)	ns
Adiponectin, µg/mL	7.4 (4.7–9.6)	6.3 (4.1–8.0)	<0.001

**Figure 1:**
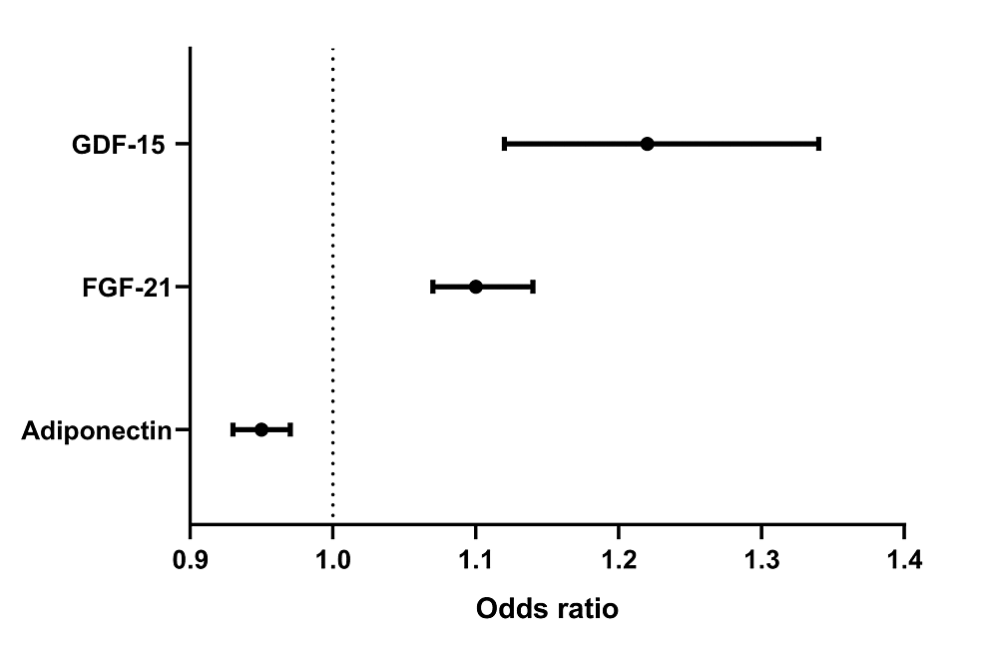
Odds ratio (Forest) plot showing the independent associations of poor sleep with three cardiometabolic markers, adiponectin, FGF-21, and GDF-15, in airline pilots FGF-21, fibroblast growth factor-21; GDF-15, growth differentiation factor-15

In the entire study cohort, significant associations were found between PSQI scores and adiponectin (Spearman’s rho = -0.46, P < 0.001), sRAGE (Spearman’s rho = -0.37, P < 0.05), FGF-21 (Spearman’s rho = 0.49, P < 0.001), and GDF-15 (Spearman’s rho = 0.43, P < 0.001). After adjustment for potential confounders in multivariable analyses, three biomarkers, including adiponectin (OR = 0.95, 95% CI = 0.93-0.97, P <0.01), FGF-21 (OR = 1.10, 95% CI = 1.07-1.14, P <0.001), and GDF-15 (OR = 1.22, 95% CI = 1.12-1.34, P <0.001), were identified as being independently associated with poor sleep in our sample of APs (Figure 1).

## Discussion

In the current preliminary cross-sectional study involving 117 APs, we discovered a 40.2% prevalence of poor sleep quality. This aligns with previously reported rates, which range from 34.9% [1] to 48.2% [14]. Notably, we found that poor sleep quality among these professionals was linked to lower plasma concentrations of adiponectin and elevated levels of FGF-21 and GDF-15. These observations remained consistent even after accounting for potential confounding variables in multivariable analysis. Although Zlokovic et al. [15] have previously reported that sRAGE in the plasma is significantly reduced after sleep deprivation, and although we also found lowered levels of this marker in our poor sleepers, the association did not persist after adjusting for potential confounders. This may suggest that the association of this molecule with poor sleep is not independent of other biomarkers, which outweighs its significance.

Adiponectin, FGF-21, and GDF-15 were the only independent predictors of poor sleep out of the fourteen plasma biomarkers of cardiometabolic health assayed in our sample of APs. Adiponectin, a major metabolism-regulating hormone with anti-inflammatory and insulin-sensitizing properties mainly released by visceral fat, has been extensively investigated in the published literature in relation to sleep deprivation and sleep disturbances. Specifically, a large body of evidence supports a decrease in circulating adiponectin concentrations in patients with OSA regardless of overweight status and obesity [16]. In addition, hypoxic stress, which has been repeatedly reported among flying personnel [17], can be at least partly responsible for the reduction of circulating adiponectin during OSA episodes [18]. Notably, adiponectin expression is circadian periodic and this adipokine has been conceptualized as a peripheral coordinator of the circadian clock in the brain and peripheral organs [19]. Since environmental synchronizers (e.g., rest-activity schedule and meal timing) are often disrupted in APs, it is not surprising that poor sleep was found to be associated with a decrease in plasma adiponectin levels.

FGF-21 is a hormone that regulates glucose and lipid metabolism, and higher levels are often associated with metabolic diseases [20]. The first description of an association between increased FGF-21 and a short sleep duration was reported by Li et al. [21] among Chinese children. More recently, Mateus Brandão et al. [22] showed that FGF-21 circulates at higher levels in metabolic conditions associated with chronic sleep-wake disruption and that serum levels of this molecule were higher after sleep loss compared with normal sleep. In an animal study, Hokari et al. [23] reported that FGF21 might influence sleep-wake regulation by inducing the production of an anti-stress agent and/or ketone bodies. Interestingly and similar to adiponectin, FGF-21 shows a diurnal oscillation and directly affects the output of the brain master clock [24]. Collectively, these results suggest that alterations in diurnal regulation of systemic energy balance may be involved in sleep impairment commonly observed in APs. In addition, GDF-15, a stress-responsive cytokine and a well-known endocrine-acting metabolic mediator [25], has been shown to promote daytime-restricted anorexia [26]. Although sleep time did not appear to be associated with GDF-15 in older adults and no association with OSA was seen, Olszowka et al. [27] reported that elevated levels of this molecule were associated with excessive daytime sleepiness and morning tiredness in a large cohort of patients with chronic coronary syndromes. In addition, spending more time in sedentary behaviors was associated with higher GDF-15 levels among those less active [27]. Considering that the occupational demands of APs can contribute to physical inactivity, further studies on the relationship between poor sleep and sedentary behaviors in this occupational group are warranted.

This study has several limitations that should be considered. First, we did not collect objective sleep quality metrics, as these were beyond the scope of standard occupational medicine consultations. Instead, we relied on the PSQI, a self-reported questionnaire. Although this method is subjective, the PSQI has demonstrated reliability and effectiveness in measuring sleep quality, with a diagnostic sensitivity of 89.6% and specificity of 86.5% for sleep disturbances [13]. Second, our sample's sex distribution accurately reflected the broader demographic of APs, but the lack of female participants limits the generalizability of our results to women. This underscores the need for more comprehensive research involving a larger and more diverse sample size to confirm and extend our conclusions. Third, we were unable to systematically collect data on other potential confounding factors, such as alcohol use or levels of physical activity. Lastly, our study was cross-sectional, which allows us to identify relationships but not to definitively establish prediction or causation between observed biomarkers and cardiometabolic risk in APs. Given these limitations, our results must be interpreted with caution and validated through future longitudinal studies, which should include more comprehensive cardiovascular assessments and more diverse participant demographics.

## Conclusions

Despite the limitations of our study, we observed that poor sleep quality among APs might be linked to lower adiponectin and elevated FGF-21 and GDF-15 levels in plasma. These findings suggest a potential influence of these cardiometabolic biomarkers on sleep quality and duration in this professional group. However, the exact nature and extent of this impact warrant further investigation. Longitudinal studies are also essential to better understand the role of these biomarkers in mediating the relationship between sleep disturbances and increased cardiometabolic risk among APs.
